# Mixed Infections

**Published:** 1913-09

**Authors:** I. Walker Hall

**Affiliations:** Professor of Pathology, University of Bristol; Pathologist, Bristol Royal Infirmary.


### MIXED INFECTIONS.

BY

I. WALKER HALL, M.D.,

*Professor of Pathology, University of Bristol; Pathologist, Bristol Royal Infirmary.*

---

MIXED bacterial infections are responsible for many puzzling groups of symptoms. The difficulties of diagnosis are increased because at one time there is a summation of effects, and at another the secondary results are more or less overshadowed by the predominant organisms. To take the case of tuberculous infections: the exudative pneumonia which occurs is generally the result of a secondary invading pneumococcus; the exudates in tuberculous pleurisy are almost always pneumococcal at first, the pneumococcus dying out later and leaving the fluid sterile; the hectic fever is probably due to the action of other organisms.

A mixed infection, however, need not necessarily be purely bacterial. Protozoal lesions may be altered by the entrance of pyogenic cocci, and some of the filtrable viruses act in concert with schizomycetes.

The primary organisms may damage the skin or mucous membranes, and so provide an easy entrance for the secondary organisms, as in tuberculosis, typhoid, dysentery or variola; or the primary organisms may damage the general body resistance, and so favour the action and spread of secondary organisms. The frequency of pneumococcal infection in typhoid fever and erysipelas illustrates the latter type.

In the spread of the mixed lesions certain fairly definite changes may be expected. The associated organisms may remain localised, as in the case of tuberculous skin lesions infected later with staphylococci. Or both types of organisms may be generalised, as in combined tuberculous and strepto-coccal infections, typhoid and para-typhoid, etc. Or the primary factor remains localised, and the secondary one becomes generalised. This occurs in the secondary infections of measles, scarlet fever and smallpox, in influenzal conditions with a secondary streptococcal manifestation in the exudates, in the streptococcal lesions occurring during diphtheria, etc. Or the primary agent may become generalised, and the secondary organism remain localised, the most familiar instance of a very long list being the localisation of the staphylococci and streptococci of a tuberculous cavity, while the tubercle bacillus extends into distant parts.

These features are easily demonstrable. Less apparent but equally important is the condition of sub-infection or sub-intoxication in relation to additional infection. The raids of bacteria present in the alimentary and genito-urinary cavities into the systemic spaces are not always unsuccessful. Now and again a few organisms remain undestroyed, manage to live at starvation rates upon the organic secretions, and constitute what has been termed " latent " bacterial action. They may, however, do more than this. Existing comfortably, without much propagation, they may bring about a condition of doubtful sub-infection, that is to say, a stage of invasion of the tissues which is neither a success nor a failure—a stalemate. On the other hand, the metabolic products of the bacteria, or the action of the bacteria upon protein materials, may depress gradually the nuclear activities of the whole body, and thus convert the doubtful local sub-infection into a definite one. In such a case the individual does not present any noteworthy symptoms. He may, however, fall an easy prey to some epidemic disease and receive the treatment for that disease, whereas he is really suffering from a mixed infection, and will not be cured entirely before both exciting agents have been dealt with.

A symbiosis of this kind calls for more than one method of attack. It is not simply a haphazard association of two or more different organisms situated in separate foci and acting individually. There is generally an interaction, and in some cases a combined action. The presence of staphylococci in a tuberculous focus leads to an increased virulence on the part of the tubercle bacillus; the bacillus typhosus acts more intensely when it is associated with the streptococcus. When the bacillus coli and the streptococcus are present under conditions of a raised acidity the streptococcal influence is depressed, while a lowered acidity, or an alkalinity, allows the streptococcus to gain the upper hand.

It is obvious, therefore, that during the course of mixed infections the bacteria themselves undergo changes which may modify appreciably the ordinary progress towards recovery. Recent investigations have shown that parasites possess very real and efficient powers of adaptability and mutation. There appears to be a continuous adaptation on the part of the micro-organism to the resisting processes of the cell nuclei, as well as to any chemio-therapeutic substances artificially introduced into the tissues. The adaptability and mutative changes naturally become more complex when several interacting agents are concerned.

The diagnosis of mixed infections from the bacteriological standpoint is fraught with many pitfalls. In an apparently pure infection a second micro-organism, or virus, may be present. When two types of bacteria are isolated from pus or other material, it is important to determine their pathogenicity. When more than two organisms are present their relative dependence and altered virulence have to be considered. To this end it is not sufficient simply to demonstrate morphological or cultural characteristics. Their behaviour when placed in the tissue fluids of the host and their defence against the forces of phagocytosis call for investigation. In other words, the resistance of the host requires to be examined. Two cubic centimetres of the serum of the patient should be subjected to ordinary and absorption tests for the content of agglutinins, precipitins and, in some cases, opsonins; while, in addition, the identification of specific protective ferments by clinical methods and estimations of the combining powers of the anti-bodies by serological procedures may be indicated. Routine blood counts are also helpful.

The treatment of these infections is not always straightforward. There are but few bacteria which lay down their arms at the first sound of battle, or even fall victims to one kind of ammunition. Diphtheria and tetanus bacilli are made less harmful by the use of antitoxins, but they are not thereby rendered incapable of producing more toxin. In the former instance the simultaneous use of a bacterial emulsion or a solution of lipoid material may bring about the more rapid disappearance of the organism; in the latter, the future may reveal the necessity of supplementing the serum injections by a bacterial emulsion composed of the organisms which furnish the symbiotic conditions necessary for the propagation of the tetanus bacillus, with the view to the removal of the assisting organism, and a consequent inhibition of the growth of the tetanus rods.

In some cases it is best, perhaps, to aim at the removal of one organism alone, and to leave the other one to natural processes. The injection of artificial serum containing lysins, opsonins, antitoxins and antiferments constitutes one of the best of measures. Unfortunately, this can be used only for a short period, and for the tiding of the patient over difficult crises. The dangers associated with family and individual idiosyncrasies materially restrict its use. Recourse must be had to the injection of bacterial emulsions with the view to stimulate the tissues to a better defence. Of late, however, the *sensitised* emulsion, or vaccine, has taken its place as a better means of treatment. A sensitised emulsion combines the properties of the serum and the emulsion. The bacteria are treated with a corresponding artificial serum, and during their contact with the contained antibodies they become altered in such a way that upon subsequent injection they stimulate the body cells to form more suitable protective substances, as well as serve as the means of conveying the antibodies of the serum to the tissues. The sensitised emulsion has further advantages in that it is less irritating locally than the ordinary emulsion, and that much larger doses of the micro-organisms may be given. It has also valuable prophylactic properties, and merits a routine use prior to operations and to childbirth. The sensitisation is generally carried out with horse or other animal sera, but now and then it has proved advantageous to use the patient's own serum or fluid exudates. In gonococcal, influenzal and similar infections such a method is almost imperative.

Another combination which the future may bring into vogue is the association of serum, or bacterial emulsion, with drug therapy. The action of pneumococcal serum is enhanced by the simultaneous use of quinine derivations, such as ethylhydrocuprein. Serums associated with cerebro-spinal conditions have proved more potent in connection with oleic and boric compounds. Anthrax anti-serum has been augmented by the administration of salvarsan. Tuberculin injections have proved more beneficial when given in connection with solutions of lecithin. Antityphoid measures have recently been supplemented by a combination of metallic solutions. Further experimentation is necessary before these methods obtain general application ; but their mention is perhaps of value in order to emphasise the necessity of treating bacterial lesions by combined and not by unified treatment. The bacterium learns to defy the stock cartridge in a few generations, that is in a few hours, and can laugh accordingly at the ineffectual attacks of the subsequent "pin-pricks."

Whether the treatment is directed against an individual bacterium or against two or more at the same time, the most important factor in the result is the estimation of the extent of adaptation manifested by the infecting agent. Is the originally predominant organism still the chief offender, or has another one taken its place ? Has the one or the other altered its characteristics in such a way as to render the emulsion, serum, sensitised vaccine or drug of little avail ? These are questions which call for an answer in every case. At all events, when the treatments appear to fail, the method employed should not be regarded as ineffectual until an attempt has been made to provide the necessary reply.

In mixed infections also the factor of previous immunisation plays a part. Subsequent inoculations may show that the rate and mode of reaction have been altered. The production of protective bodies may be more rapid and abundant, or they may not occur at all. On the other hand, there may be an increased susceptibility. The observation that when an immunity to bacillus coli is induced the injection of dead staphylococci will raise the agglutinating power of the serum for bacillus coli, illustrates the problem looked at from the standpoint of mixed action. Reactions like these appear to border upon anaphylactic phenomena. Little as we yet know of their biological significance, their recognition impresses upon us the futility of injecting unknown stock emulsions without due consideration of the factors already determined. Discredit, personal and general, will not be avoided unless the treatment of bacterial infections by anti-bacterial measures is undertaken with a fuller knowledge of the after-effects than is now deemed necessary.

### A CASE OF CERVICAL RIB.

BY

RUPERT WATERHOUSE, M.D., M.R.C.P.,

